# Dynamic coupling modelling and application case analysis of high-slip motors and pumping units

**DOI:** 10.1371/journal.pone.0227827

**Published:** 2020-01-30

**Authors:** Ziming Feng, Qingyang Ma, Xiaolei Liu, Wei Cui, Chaodong Tan, Yang Liu

**Affiliations:** 1 School of Mechanical Science and Engineering, Northeast Petroleum University, Daqing, Heilongjiang, China; 2 School of Engineering, University of Glasgow, Glasgow, Scotland, United Kingdom; 3 School of Petroleum Engineering, China University of Petroleum, Beijing, China; Huazhong University of Science and Technology, CHINA

## Abstract

To solve the issues and difficulties in the high-coupling modelling of beam pumping units and high-slip motors, external characteristic experiments of high-slip motors were performed where the external database and characteristic correlation equations of the motors were obtained through data regression analysis. Based on the analysis of the kinematics, dynamics and driving characteristics of the beam pumping unit, a fully coupled mathematical model of a motor, pumping unit, sucker rod and oil pump was established. The differential pumping equation system of the pumping unit used a cyclic iteration method to solve the problem of high coupling among the motor, pumping unit, sucker rod and the pumping pump. The model was verified by experimental data of field l pumping wells. Theoretical calculations and experimental tests showed that the soft characteristic of the high-slip motor can reduce the peak suspension load of the sucker rod, peak net torque of the gearbox and peak power of the motor. In addition, the results show that the soft characteristic can also decrease the high-frequency fluctuation of the motor power curve and the torque curve of the gearbox. The high-slip motor can improve the smoothness and safety of the pumping well system.

## Introduction

The high-slip motor drives the beam pumping unit with several advantages. The starting torque is large, and the peak torque of the pumping unit can be reduced, which is suitable for loading the start of the pumping unit [1]. Therefore, the oil well adopts a high-slip motor that can reduce the capacity of the power supply system, such as the motor and the transformer, decrease the energy consumption, and improve the power factor. In addition, the high-slip motor has soft mechanical characteristics which adapt to the cyclic alternating load of the beam pumping unit, making the pumping unit system run smoothly and increasing the service life of the equipment [2].

In 1963, Gibbs [3] first established a one-dimensional wave equation for the sucker-rod string. In addition, Doty and Schmidt [4] established two-dimensional wave equations for sucker-rod string and liquid columns. In 1988, Yu [5] established a three-dimensional wave equation of sucker-rod string, liquid column and tube strings, increasing the calculation accuracy of the suspended point load of the pumping unit. However, these studies did not consider the effect of motor speed change on the dynamic performance of the pumping unit system. When a high-slip motor is used to drive the pumping unit, the effect of the motor slip rate on the system was not considered, generating a large prediction error.

API standards do not consider inertia effects and should not be expected to give correct results when high-slip or ultrahigh-slip motors are used. The high-slip motors can use inertia effects to decrease equipment loading, particularly on the gearbox and the motor itself. To precisely compute the dynamic load and the torque of the sucker-rod pumping with high-slip motors, Gibbs [6] adapted a 10° crank-angle grid that is finer than the 15° interval specified by the standard API method.

In 1990, Tripp et al. [7] used Doty and Schmidt`s mathematic model to predict the performance of the sucker-rod pump. Doty and Schmidt considered motor slip and rotating and articulating inertia and achieved results indicating that 98% of the actual peak rod-loads should not be more than 6% above the calculated value. In 1991, Yao [8] et al. established an equivalent mechanical model and a differential equation of motion for the dynamic coupling of a high-slip motor and a pumping unit and launched a wave equation for the downhole sucker rod system, but they did not consider the effect of tubing and liquid column vibration on the suspension load. Consequently, the calculation has errors. By taking the minimum period load coefficient as the target, Shen et al. [2] proposed the principle and method of matching the optimal slip of the motor.

In 1996, Qi et al. [9] proposed an equivalent dynamic model of the beam pumping unit that uses the moment of inertia of the crank and the connecting rod to the crankshaft. It solved the problem of coupling the motor and the pumping unit but did not explain how to confirm the problem of motor slip. Dong [10] and Xue [11] in 1996 and in 2002, respectively, used the interpolation method and the McMack method to solve the wave equation, respectively, and considered the motor slip rate and the moment of inertia of the crank and the connecting rod. However, the application of these methods to solve the problem of high coupling between the pumping unit and the drive motor is very complicated. In 2001, Dong et al. [12] introduced a three-dimensional vibration model of the sucker rod column to the dynamic coupling model of the high-slip motor and the pumping unit.

In recent years, some researchers continued to conduct on research work of a sucker-rod pump. In 2015, Xing et al. [13] combined the belt transmission efficiency slip model and one-dimensional wave equation to improve the optimise speed and accuracy of the dynamic performance of the sucker-rod pump. In 2016, Xing [14] established a model of the longitudinal vibration and flexural coupling of the sucker-rod pump and calculated its column mechanics by ANSYS software. In 2016, Lao et al. [15] establish a lateral force coupling model between buoyancy and the tube in the inclined well, revealing how buoyancy affects the dynamic performance of the sucker-rod. In 2018, Feng et al. [16] established a three-dimensional dynamic model of the rod-tube-liquid and analysed the dynamic performance of the sucker-rod pump. The simulation results are in good agreement with the experimental results with high calculation precision. Li et al. [17] presented an improved sucker-rod pump model with a fluid-solid coupled model with surface equipment, rod vibration, plunger motion, and fluid flow. In their numerical method, the motor rated-slip ratio was considered, but the motor slip was a constant value that could not present the real change rule of motor slip and was not suitable for the high-slip and superhigh-slip motors.

Variable speed drive (VSD) is a new technology that improves the performance of the sucker-rod pump by changing the speed of the prime motor and has the same function as to the high-slip motor. In 2011, Sam et al. [18] used the depth data of the downhole surface and the VSD to optimise pump efficiency. In 2015, Elmer [19] maintained the upstroke speed by reducing the downstroke speed to improve pump efficiency by VSD. Carpenter [20] proposed VSD technology to detect the condition and improve oil output based on the coefficient of discharge of the pump. In 2016, Burgstaller proposed a method to prevent pumping and optimise production based on VSD [21]. Clarke [22] applied VSD technology for 550 oil wells and discussed in detail the causes of failure of some wells. In 2016, Dong et al. [23] established a variable frequency speed optimisation method for beam pumping units using a one-dimensional wave equation that does not consider the effect of the liquid column. In 2017, Alwazeer et al. [24] used their VSD technology to optimise production by keeping the hydrodynamic surface slightly above the surface of the pump. In 2018, document [25] proposed the use of VSD technology to improve the effects of gas. Document [26] uses VSD technology to control the pumping speed to achieve the maximum production of the oil well. Ferrigno [27] combines the intelligent well controller technology with the telemetry Systems technology to achieve maximum throughput and efficiency goals for 50 wells.

The presented literature used one-dimensional, two-dimensional and three-dimensional wave equations and the motor differential equation to solve the influence of inertial load on the performance of the pumping unit system. However, because the simplified equation of the external characteristics of the motor cannot truly reflect the slip rate of the high-slip and the ultra-high-slip motor, the calculation result still has a relatively large error.

To solve the problem of power coupling between high-slip motor and pumping unit, first, the external characteristics of the high-slip motor are obtained through experiments, and the calculation functions of slip, torque, power, power factor, current and efficiency of the motor were obtained using experimental data. Second, combined with the kinematics equation of the pumping unit and the three-dimensional wave equation of the sucker rod, and with the oil pump as the boundary condition, the fully coupled mathematical model of the high-slip motor-pumping unit-sucker rod-oil pump was established and calculated using Visual Basic® V6.0 software. Third, by calculating the performance parameters of the pumping well system using a multi-port high-slip motor and comparing with the measured data, the accuracy of the fully coupled mathematical model of the pumping unit system and its solving method are verified. Lastly, the experimental study compared the dynamic effects of a low-slip motor (less 3%) and high-slip motor (about 10%) on a pumping well system. Experiments showed that the high-slip motor can effectively reduce the peak load and the volatility of the pumping unit, benefiting the safe operation of the beam pumping unit.

## Experiments on external characteristics of high-slip motors

The slip rate of an ordinary asynchronous motor is less than 3%, while the slip rate of a high-slip motor is generally about 10%, but some motors have a slip rate of more than 30%. By relying on the motor performance data, speed can be used to infer gearbox torque, unit balance, motor load, qualitative dynamometer cards, and power consumption.

Solving the dynamic coupling solution of the pumping unit and the high-slip motor requires accurate external motor characteristic data. Based on the motor performance data, speed can be used to infer gearbox torque, unit balance, motor load, qualitative dynamometer cards, and power consumption. However, the data provided by general motor manufacturers are not ideal [28], and the motor load rate is relatively low. Data below 30% are generally not available, but the transient load rate of the motor for pumping units is typically between 0% and 100%. Therefore, this paper first performed experimental research on the external characteristics of the motor, including the locked-rotor test, no-load test, loading test, and efficiency test.

**Fig 1:**
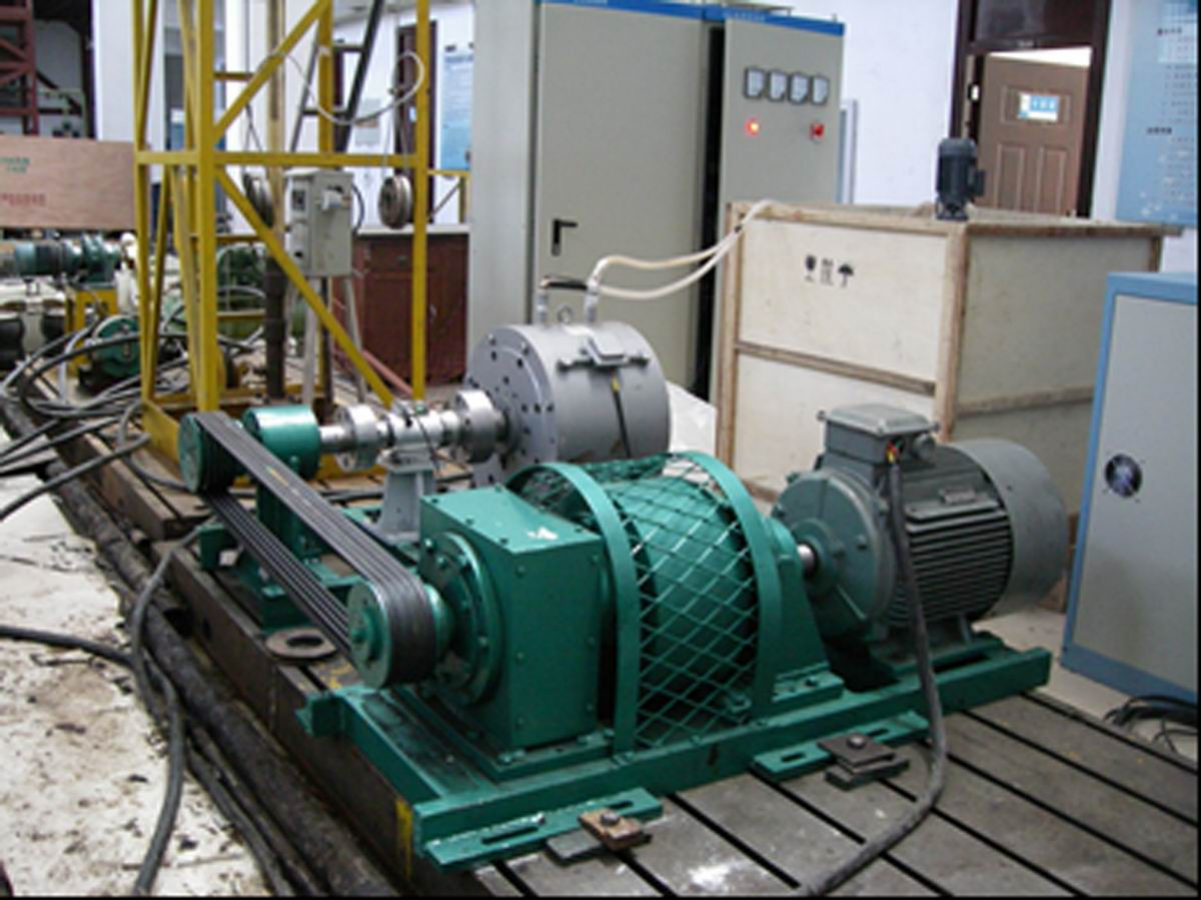
Experimental apparatus for external characteristics of the motor.

The experimental procedure of the external characteristics of the motor is displayed as follows (see Fig 1):

1. The motor to be tested was installed on the test rig and was coaxial with the eddy current dynamometer. Otherwise, the test result has a great influence. Before loading the test, it is confirmed whether the motor is unloaded normally.
2. Check whether the water-cooling device of the eddy current dynamometer is turned on.
3. The motor under test runs in the state of the motor, keeping the rated voltage constant. The speed is kept as high as possible.
4. Adjust the eddy current dynamometer controller to gradually increase the current of the tested motor from zero to the rated value and adjust the output voltage of the regulator to maintain the rated voltage.
5. Gradually increase the loading rate so the motor output power varies from 0% to 100% of rated power.
6. Record parameters such as voltage, current, power, power factor, speed, output torque, and output power during motor operation.
7. Analyse the test data and plot the external characteristic curve. Regress the external characteristic function to establish a functional equation of slip, power factor, input current, input power, efficiency, and output power.

**Fig 2:**
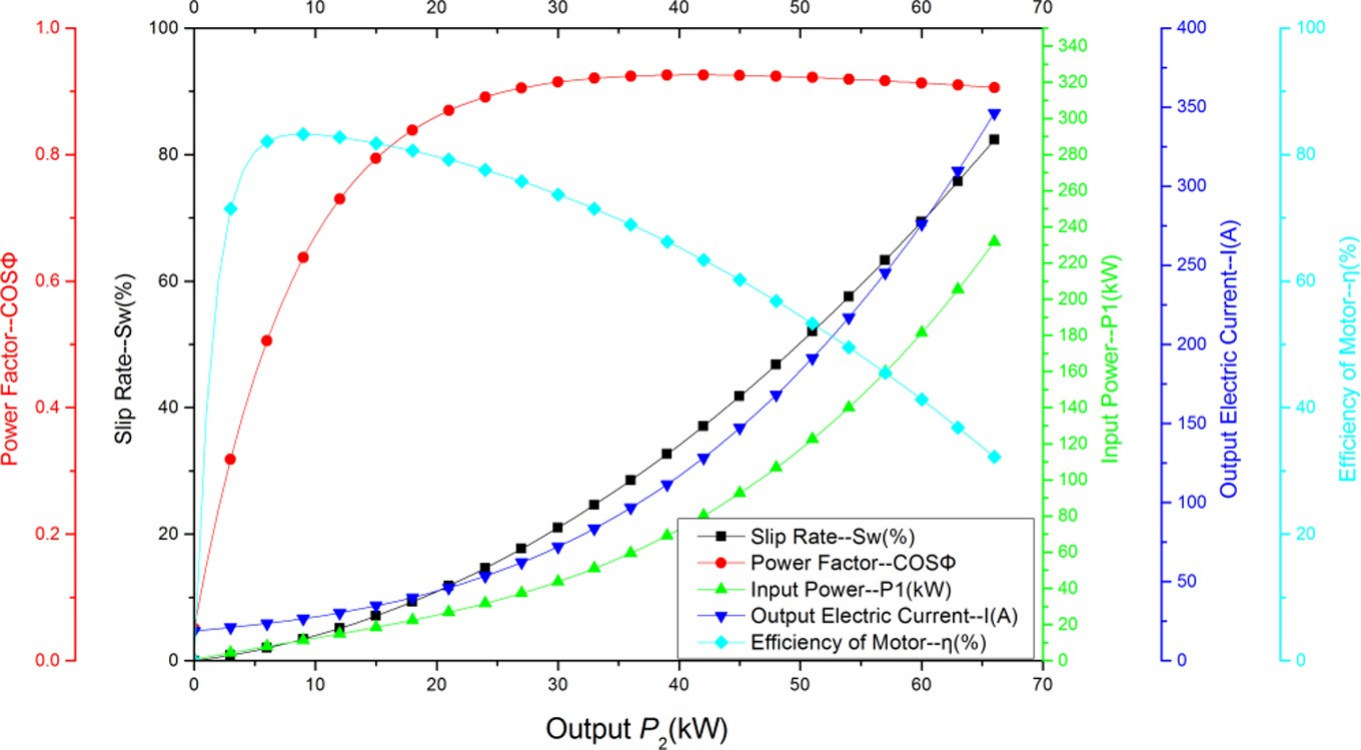
YCCH225-6 drive motor operating characteristics.

The external characteristic equations of the tested motor (YCCH225-6/22kw) are presented in Eqs (1)–(6). Fig 2 shows the complete external characteristic curve of the high-slip motor.

Power factor:

$$\cos\phi {\left(p_{2}\right)}_{22\mathrm{kw}‐\mathrm{YCCH}}=-0.8988\times 0.889p_{2}-0.00001p_{2}^{2}+0.95$$ (1)

Input power:

$$p_{1}{\left(p_{2}\right)}_{22\mathrm{kw}‐\mathrm{YCCH}}=0.0008p_{2}^{3}-0.0194p_{2}^{2}+1.2958p_{2}+0.7$$ (2)

Input electric current:

$$I_{1}{\left(p_{2}\right)}_{22\mathrm{kw}‐\mathrm{YCCH}}=0.0008p_{2}^{3}+0.0117p_{2}^{2}+0.7064p_{2}+18.761$$ (3)

Efficiency of the motor:

$$\eta {\left(p_{2}\right)}_{22\mathrm{kw}‐\mathrm{YCCH}}=84.5-84.5e^{\left(-p_{2}/1.6\right)}-0.012p_{2}^{2}$$ (4)

Slip ratio:

$$s_{w}{\left(p_{2}\right)}_{22\mathrm{kw}‐\mathrm{YCCH}}=0.0152p_{2}^{2}+0.2445p_{2}$$ (5)

Mechanical characteristic:

$$n=-3.12\times 10^{-8}T^{3}+2.58639\times 10^{-5}T^{2}-0.75T+1000$$ (6)

Where

*p*_2_—output power of the motor, kW;

*s*_*w*_ *—*slip ratio;

*p*_1_—input power of the motor, kW;

*I*_1_—input electric current of the motor, A;

cos*φ—*power factor of the motor;

*η*—efficiency of the motor.

## Coupling model of beam pumping unit and drive motor

### A. Beam pumping unit variable speed kinematics

#### a. Suspended point displacement

The relationship between the suspension displacement of the beam pumping unit and the crank angle is a geometric relationship. The suspension displacement depends only on the geometrical dimensions of the four-bar linkage of the pumping unit and has no relationship with the angular velocity of the crank changes.

$$\delta_{i}=\pm (\psi_{b}-\psi )$$ (7)

$$S_{c}=A\delta_{i}$$ (8)

Eq 7 and Eq 8 only reflect the relationship between the suspension displacement and the crank angle and cannot reflect the association between the suspension displacement and the time. The relationship between the crank angle and time is presented as follows:

$$t=\int \frac{d\theta }{\omega (\theta )}$$ (9)

The relationship between the crank rotational speed and the crank angle *ω*(*θ*) is confirmed to determine the correspondence between the crank angle *θ* and the time *t*.

#### b. Suspended point velocity

The angular velocity of the walking beam is the following:

$$\omega_{b}=\frac{d\delta_{i}}{dt}=\omega \frac{d\delta_{i}}{d\theta }$$ (10)

The suspension speed equation at *ω* is equal to a constant, there is no change in form, and it can still be used in variable speed motion. However, *ω* in Eq (10) is no longer constant. It is a variable *ω*(*θ*) that varies with the crank angle. By knowing the law of change of *ω*(*θ*), we can determine the variation law of the speed of the suspension point.

#### c. Suspended point acceleration

The angular acceleration of the beam swing is the following:

$$\varepsilon_{b}=\frac{d\omega_{b}}{dt}=\varepsilon_{b1}+\varepsilon_{b2}$$ (11)

The angular acceleration of the angle of the walking beam is composed of two parameters. The first parameter *ε*_*b*1_ is equivalent to the natural angular acceleration of the beam determined by the characteristics of the four-bar linkage itself, while the crank angular velocity is constant (*dω*/*dθ* = 0). The second parameter *ε*_*b*2_ is the additional angular acceleration generated by the instantaneous angular velocity change of the crank angle.

$$\varepsilon_{b1}=\frac{KR}{CP}\omega_{c}{}^{2}\frac{\mp \sin\beta \cos\alpha \sin\psi -\frac{R}{C}\sin\alpha \cos\beta \sin\theta_{2}}{\sin^{3}\beta }$$ (12)

$$\varepsilon_{b2}=\omega_{b}\frac{d\omega_{c}}{d\theta }=\frac{\bar{TF}}{A}\varepsilon$$ (13)

$$\varepsilon =\frac{d\omega_{c}}{dt}=\omega_{c}\frac{d\omega_{c}}{d\theta }$$ (14)

$$a_{c}=A\left(\frac{KR}{CP}\omega_{c}{}^{2}\frac{\mp \sin\beta \cos\alpha \sin\psi -\frac{R}{C}\sin\alpha \cos\beta \sin\theta_{2}}{\sin^{3}\beta }+\frac{\bar{TF}}{A}\omega_{c}\frac{d\omega_{c}}{d\theta }\right)$$ (15)

Where

*δ*_*i*_— angular displacement of the beam swing,°;

*δ —*maximum angular displacement of the beam swing,°;

*θ*—crank rotor angle,°;

*ε*_*b*_—angular acceleration of the swing angle of the beam, m/s^2^;

*ε*_*b*1_ *—*natural angular acceleration of the swing angle of the beam, m/s^2^;

*ε*_*b*2_—additional angular acceleration of the swing angle of the beam, m/s^2^;

*ε*—crank angular acceleration, m/s^2^;

*a*_*c*_— suspension acceleration, m/s^2^;

*ω*_*b*_— angular velocity of the swing angle of the beam, rad/s;

*ω*_*c*_— crank rotation angular velocity, rad/s;

*A*— forearm length, m;

*K*—base bar length, m;

*R*—crank radius, m;

*C*—beam rear arm length, m;

*P*— connecting rod length, m;

*β*— transmission angle,°;

*α*— the angle between the crank and the connecting rod,°;

*ψ*— angle between *c* and *k*,°;

*θ*_2_—angle between the crank and the base bar,°;

*θ*— crank angle,°;

$\bar{TF}$— torque factor.

### B. Suspended point dynamics model

#### a. Three-dimensional wave equation

Because the tubing of pumping well in China is not anchored, it is necessary to comprehensively consider the three-dimensional vibration of the rod string, liquid column and pipe string [5]. The partial differential equations of three-dimensional vibration in Eq (16) can calculate the displacement and load of sucker rod at arbitrarily axial positions.

$$\left\{ \begin{array}{l} \rho_{r}A_{r}\frac{\partial v_{r}}{\partial t}=\frac{\partial f_{r}}{\partial x}+\rho_{r}A_{r}g\left(1-\frac{\rho_{f}}{\rho_{r}}\right)-F_{r}\\ E_{r}A_{r}\frac{\partial v_{r}}{\partial x}=\frac{\partial f_{r}}{\partial t}\\ \rho_{r}(A_{h}-A_{t})\frac{\partial v_{t}}{\partial t}=\frac{\partial f_{t}}{\partial x}\rho_{t}g(A_{h-}A_{t})-F_{t}\\ E_{t}(A_{h}-A_{t})\frac{\partial v_{t}}{\partial x}=\frac{\partial f_{t}}{\partial t}\\ \rho_{f}(A_{t}-A_{r})\frac{\partial v_{f}}{\partial t}=-(A_{t}-A_{r})\frac{\partial p_{f}}{\partial x}-F_{f}+\rho_{f}(A_{t}-A_{r})g\\ E_{f}\frac{\partial v_{f}}{\partial x}=-\frac{\partial P_{f}}{\partial t} \end{array}\right.$$ (16)

Differential Eq (16) described the mathematical model of the rod string, liquid column and tube string three-dimensional fluctuations to solve the stress and displacement at arbitrary positions of the sucker rod.

Where

*v*_*r*_— rod velocity, m/s;

*v*_*t*_ *—*tube velocity, m/s;

*v*_*f*_—liquid column velocity, m/s;

*E*_*r*_—rigidity of the rod, N/m^2^;

*E*_*t*_—rigidity of the tube, N/m^2^;

*E*_*f*_—rigidity of oil, N/m^2^;

*A*_*r*_—sectional area of the rod, m^2^;

*A*_*t*_—inner area of the tube, m^2^;

*A*_*h*_—outer area of the tube, m^2^;

*ρ*_*r*_—density of the rod, kg/m^3^;

*ρ*_*f*_— liquid density, kg/m^3^;

*ρ*_*t*_— density of the tube, kg/m^3^;

*F*_*r*_— resistance met with the rod, N/m^2^;

*F*_*t*_— resistance met with the tube, N/m^2^;

*F*_*f*_—assistance met with the liquid column, N/m^2^;

*P*_*f*_—pressure of the liquid, N/m^2^;

*g*— gravity acceleration, m/s^2^.

#### b. Damping force calculation

The damping force of the sucker rod is divided into three parts: viscous damping *F*_*rf*_ between the liquid and the surface of the sucker rod per unit length; viscous damping force *F*_*cf*_ between the liquid and the surface of the rod coupling; friction between the tubing and the sucker rod per unit length. Among them, although the friction between the rod and tube is not ignored, it is not considered in this paper. The reason is that it depends on factors such as the inclination of the well, and the inclination value is difficult to obtain.

The resistance of the sucker rod *F*_*r*_ is as follows:

$$F_{r}=b_{r}v_{r}-b_{fr}v_{f}$$ (17)

The resistance of the pumping pipe *F*_*t*_ is as follows:

$$F_{t}=b_{t}v_{t}-b_{fr}v_{f}$$ (18)

The resistance *F*_*f*_ of the liquid column is as follows:

$$F_{f}=-\left[F_{r}+F_{t}\right]$$ (19)

$$b_{r}=12\pi \eta \left[\left(0.2+0.39\frac{D_{r}}{D_{t}}\right)+\frac{2.197}{24}{\left(\frac{D_{c}}{D_{t}}-0.381\right)}^{2.57}\frac{{\left(D_{c}/D_{r}\right)}^{2}-1}{1/D_{r}}\right]\frac{D_{r}}{D_{t}-D_{r}}$$ (20)

$$b_{fr}=12\pi \eta \left[1+667.08{\left(\frac{D_{c}}{D_{t}}-0.381\right)}^{2.57}\frac{{\left(D_{c}/D_{r}\right)}^{2}-1}{1/D_{r}}\right]\frac{D_{r}}{D_{t}-D_{r}}$$ (21)

$$b_{t}=12\pi \eta {\left(0.2+0.39\frac{D_{r}}{D_{t}}\right)}_{r}$$ (22)

$$b_{ft}=12\pi \eta \cdot \frac{D_{r}}{D_{t}-D_{r}}$$ (23)

Where

*D*_*r*_—the diameter of the rod, mm;

*D*_*t*_ *—*the inner diameter of the tube, mm;

*D*_*c*_— Sucker rod coupling diameter, mm;

*η*—Hydrodynamic viscosity, N/ m^2^s;

*π*—Constant, 3.14.

#### c. Damping force calculation

The torque of the crankshaft includes the drag moment *T*_*n*_ of the polished-rod load on the crank and the active moment $iT_{d}\xi_{m}^{m}$ from the drive motor. The equivalent moment of inertia *J*_*p*_ is used to represent the moment of inertia of the whole pumping system on the crankshaft. The total gear ratio from the motor to the gearbox is *i*, so the torque balance equation on the crankshaft is shown in Eq (24):

$$iT_{d}\xi_{m}^{m}-T_{n}=J_{p}\varepsilon =J_{p}\omega \frac{d\omega }{d\theta }$$ (24)

The drag torque on the crank is the following:

$$T_{n}=\xi_{b}^{m}\left(P-B+\frac{J_{b}}{A^{2}}\omega^{2}\frac{d\bar{TF}}{d\theta }+\frac{J_{b}\bar{TF}}{A^{2}}\omega \frac{d\omega }{d\theta }\right)\bar{TF}-M\sin\left(\theta +\tau \right)$$ (25)

The equivalent moment of inertia *J*_*b*_ is the following:

$$J_{p}=J_{p0}i^{2}+J_{p1}i_{1}^{2}+J_{p2}i_{2}^{2}+J_{p3}$$ (26)

Where

*T*_*d*_—Motor shaft output torque, kN·m;

*T*_*n*_ *—*The moment of resistance of the polished rod on the crank, kN·m;

*P*—Suspended load, kN;

*B*—Unbalanced weight of pumping unit structure, kN;

*J*_*b*_—Beam moment of inertia, kg·m^2^;

*M*—Crank balancing torque, kN·m;

*τ*—phasing degree,°;

*ξ*_*m*_—Transmission efficiency of the motor to the crankshaft;

*m*—index, when *T*_*d*_>0, *m* = 1, when *T*_*d*_<0, *m* = −1;

*J*_*p*_—Motor shaft equivalent moment of inertia, kg·m^2^;

*J*_*p*0_—Moment of inertia of each component of the drive motor on the motor shaft, kg·m^2^;

*J*_*p*1_—Moment of inertia of each component of the input shaft of the redactor, kg·m^2^;

*J*_*p*2_—Moment of inertia of each component of the intermediate shaft of the redactor, kg·m^2^;

*J*_*p*3_—Moment of inertia of each member of the crankshaft, kg·m^2^;

*i*—Total gear ratio from the motor output shaft to the gearbox output shaft;

*i*_1_—Total gear ratio of the pumping unit gearbox;

*i*_2_—Pumping gear gearbox low-speed gear ratio.

By substituting Eq (25) into Eq (24), the differential equation of crank angular velocity and crank angle is obtained, as shown in Eq (27):

$$\frac{d\omega }{d\theta }=\frac{iT_{d}\xi_{m}^{m}-\xi_{b}^{m}\left(P-B+\frac{J_{b}\omega^{2}}{A^{2}}\frac{d\bar{TF}}{d\theta }\right)\bar{TF}+M\sin\left(\theta +\tau \right)}{\omega \left(J_{p}+\xi_{b}^{m}\frac{{\bar{TF}}^{2}}{A^{2}}J_{b}\right)}$$ (27)

In the case of non-equal rotation of the crank, the net torque of the output shaft of the gearbox should be equal to the sum of the rod torque, balance torque at the crank, and moment of inertia of the various components on the crankshaft.

$$T_{nh}=T_{n}+J_{p3}\varepsilon =\xi_{b}^{m}\left[P-B+\frac{J_{b}}{A}\varepsilon_{b}\right]\bar{TF}-M\sin(\theta +\tau )+J_{p3}\varepsilon$$ (28)

Where

*T*_*nh*_—Net torque of the output shaft of the gearbox, kN·m;

*ξ*_*b*_—Transmission efficiency of four-bar linkage;

*m*—index, when $\bar{TF}>0$, *m* = −1, when $\bar{TF}<0$, *m* = 1;

The *ε* is obtained by numerically solving the differential equation of motion. Motor output torque:

$$T_{d}=\frac{1}{i\xi_{m}^{m}}\left(T_{n}+J_{p}\varepsilon \right)=\frac{1}{i\xi_{m}^{m}}\left[T_{nh}+\left(J_{p}-J_{p3}\right)\varepsilon \right]$$ (29)

Eq (26) is the motion calculus equation of the crank under non-equal rotation of the crank. The motor output shaft torque *T*_*d*_ in the equation is a function related to the rotational speed of the motor. It can be solved according to the external characteristic equation of the motor mentioned previously. The relationship between the motor output torque and the crank angular velocity and the suspension load *P* can also be obtained as a function of crank angle *θ*. Therefore, the right side of Eq (28) is a function of *ω* and *θ*.

$$\frac{d\omega }{d\theta }=f\left(\omega ,\theta \right),\;\theta =\theta_{0},\;\omega =\omega_{0}$$ (30)

The equation is a first-order nonlinear differential equation, but the functional relationship of each parameter in the equation is relatively complicated, so the numerical solution method cannot be used to solve the equation between the suspended point load and the crank angle. Usually, the crank angle of the suspension point at the bottom dead centre is the starting value of *θ*_0_, the angular velocity of the crank is first given an initial average value, and the cycle is performed in a reasonable step to obtain a stable value that meets the condition.

## Solution method for dynamic coupling equation of pumping unit and high-slip motor

When solving the displacement differential Eq (26) of pumping unit by a numerical method, it is necessary to know the variation rule of suspension load *P*_*i*_ (i.e., the light rod indicator diagram), which only considers the effect of the variation of suspension load *P*_*i*_ on the variation of crank angular velocity *ω*. In fact, changing the crank angular velocity *ω* changes the suspension velocity and acceleration, thus affecting suspension load *P*_*i*_. Therefore, it is an interactive relationship. The differential equation of the pumping unit and the rod string can only be connected through considering the motor, beam pumping unit, rod string, and downhole oil pump as one system. In this way, the issues of predicting the operating parameters of the pumping system (such as suspension load, torque of a gearbox and power of a motor) can also be solved.

The calculation procedures were designed as follows:

1. Regardless of the change of the angular velocity of the crank, the average angular velocity is calculated according to the stroke. In addition, the suspension displacement, velocity, and acceleration are obtained according to the four-bar linkage motion equation.
2. By using the displacement, velocity, and acceleration of the suspended point as the initial boundary conditions, the three-dimensional wave equation of the rod, tube, and fluid is solved, and the variation law of the suspended point load *P*_*i*_ is obtained.
3. The obtained *P*_*i*_ variation rule is substituted into Eqs (26), (27) and (28) to calculate the motor torque. According to the external characteristic Eq (6) of the motor, the transient value of the speed in one stroke of the motor is obtained. The cubic spline quotient method is used to solve the acceleration of the motor for the discrete speed data.
4. According to the transient speed of the motor within one stroke, the suspension displacement, suspension velocity, and suspension acceleration are obtained by the four-bar linkage motion equation.
5. Based on the new law of the movement of the suspension point, the three-dimensional wave equation of the rod, tube and fluid is solved again, and the variation law of the suspension load *P*_*i*+1_ can be obtained.
6. Comparing *P*_*i*+1_ to *P*_*i*_, the calculation ends while the error is within the allowable tolerances. Otherwise, the calculation starts from step 2) until the error falls into the allowed ranges.
7. According to the motor external characteristic Eqs (1)–(6), all the characteristic parameters of the motor in one stroke are solved. Compared with the experimental data, the calculation accuracy of the dynamic coupling equation between the high-slip motor and the pumping unit is analysed.

## Case simulation and verification

According to the dynamic coupling model of the beam pumping unit and the high-slip motor established in the previous section, the calculation programme is compiled using the Visual Basic® V6.0 language, and the torque test results are outputted by the pumping unit gearbox to verify the accuracy of the theoretical calculation.

Under the same working conditions, the dynamic performance of the permanent magnet synchronous motor (TNM280S-8) with a rated power of 37 kW and the high-slip motor (YCCH225-6) driving pumping unit with a rated power of 22 kW was calculated. The slip rate of permanent magnet synchronous motor is zero. The motor torque, crank angular velocity, crank angular acceleration, crank torque and suspension load were analysed. The influence of the high-slip motor on the dynamic performance of the pumping well was also analysed.

## A. Calculation parameter

**Fig 3:**
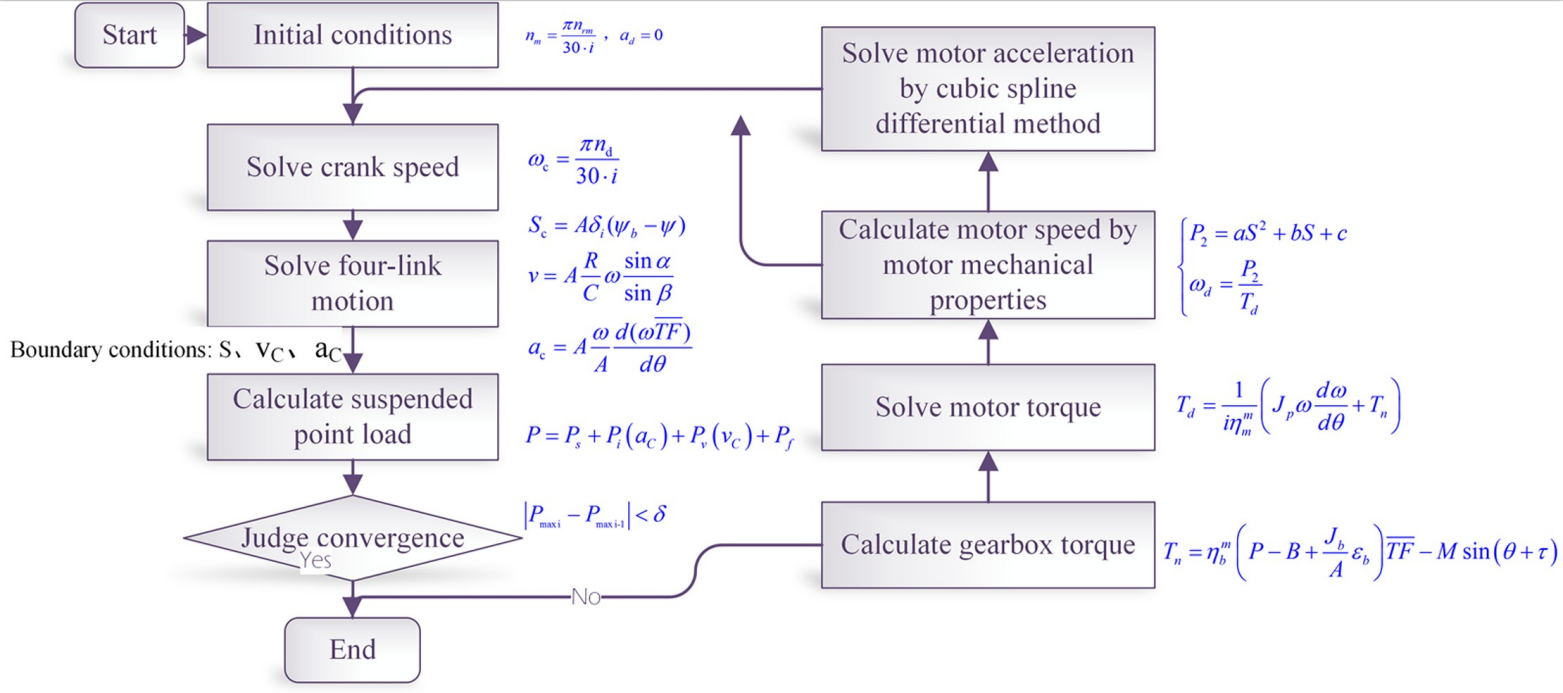
A flow chart for calculation procedures of the solution method.

The structural parameters, transmission ratio and rotation inertia of the conventional beam pumping unit CYJ10-3-37HB are shown in Table 1. The external characteristics of the high-slip motor are presented in Eqs (1)–(6) and Fig 1. The swabbing parameters of the coupling calculation are shown in Table 2. The flow chart for the calculation procedures of solution method is shown in Fig 3.

**Table 1:** Structure parameters of the pumping unit.

Pumping unit model	CYJ10-3-37HB
**Forearm length (mm)**	3000
**Rear arm length (mm)**	2400
**Connecting rod length (mm)**	3350
**Horizontal distance (mm)**	2300
**Crank radius (mm)**	1150
**Sting center height (mm)**	5290
**Crank balance radius (mm)**	750
**Transmission component**	Transmission ratio (Dimensionless)
**Motor-crankshaft**	111
**Motor shaft—belt shaft**	3.7
**Gearbox—crankshaft**	30
**Moving parts of pumping unit**	Rotation inertia (kg.m2)
**Motor output shaft**	1.39
**Gearbox input shaft**	1.29
**Gearbox intermediate shaft**	1.29
**Gearbox crankshaft**	5330
**Walking beam of Pumping unit**	2140

**Table 2:** Pumping well system pumping parameters.

Motor type	Permanent magnet Synchronous motor	TNM280S-8 (37kW)
	High slip motor	YCCH225-6 (22kW)
**Stroke (m)**		3
**Frequency of stroke (1/min)**		6
**Depth of plunger (m)**		1000
**Working fluid level (m)**		600
**Pump diameter (mm)**		57
**Rod diameter (mm)**		22
**Water percentage (%)**		90

**Fig 4:**
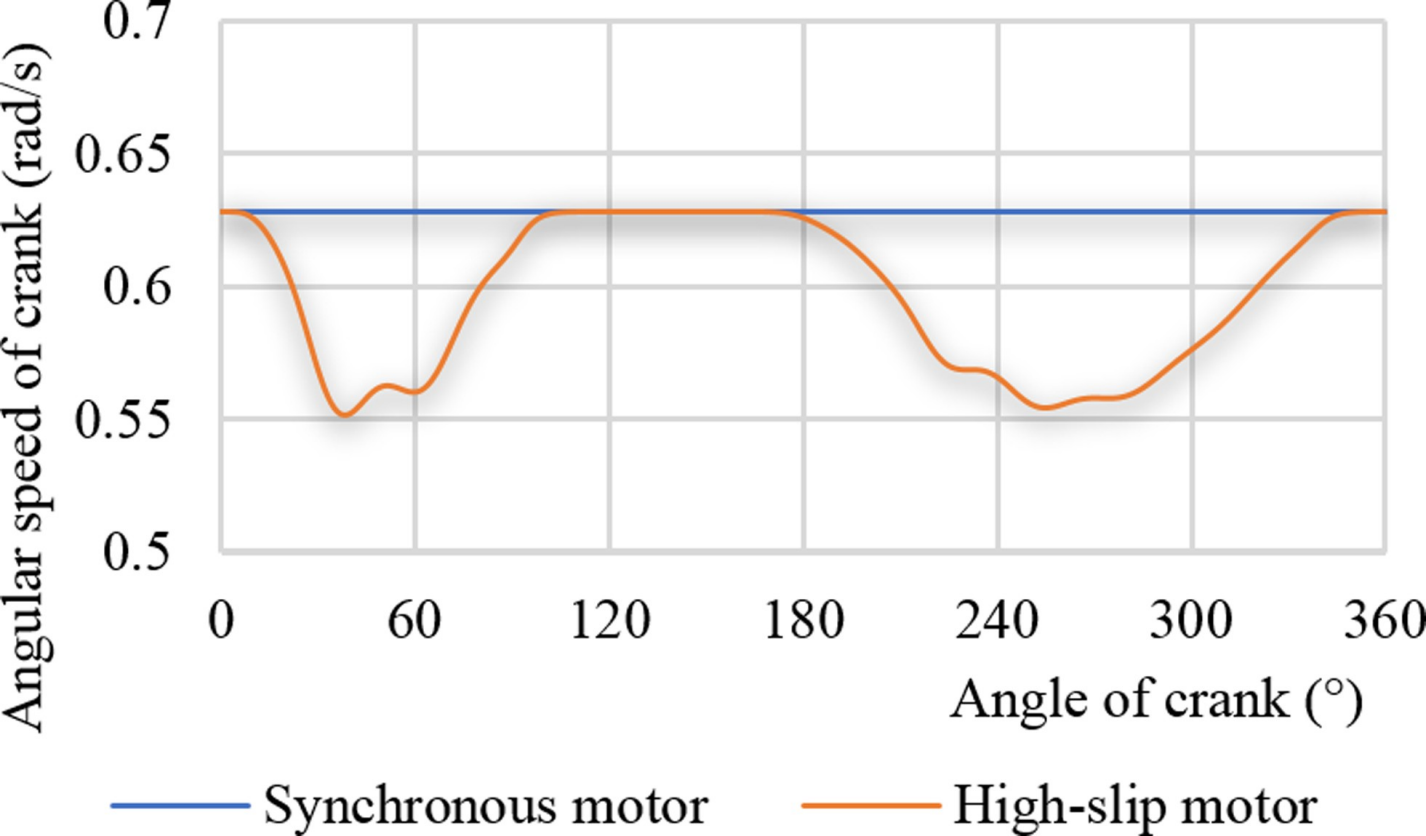
Angular velocity curves of crank.

The two curves in Fig 4 are the angular speed curves of crank under the driving conditions of the synchronous motor and the high-slip motor, respectively. When the synchronous motor is driven, the crank speed is a constant value of 0.628 rad/s. When the high-slip motor is driven, the crank speed varies from 0.553 to 0.628 rad/s. Compared with the net torque curve of the gearbox, higher net torque value occurred while smaller speed value happened corresponding to the crank, reflecting the soft characteristic of the high-slip motor, that is, the dynamic coupling characteristic of the high-slip motor and the pumping unit.

**Fig 5:**
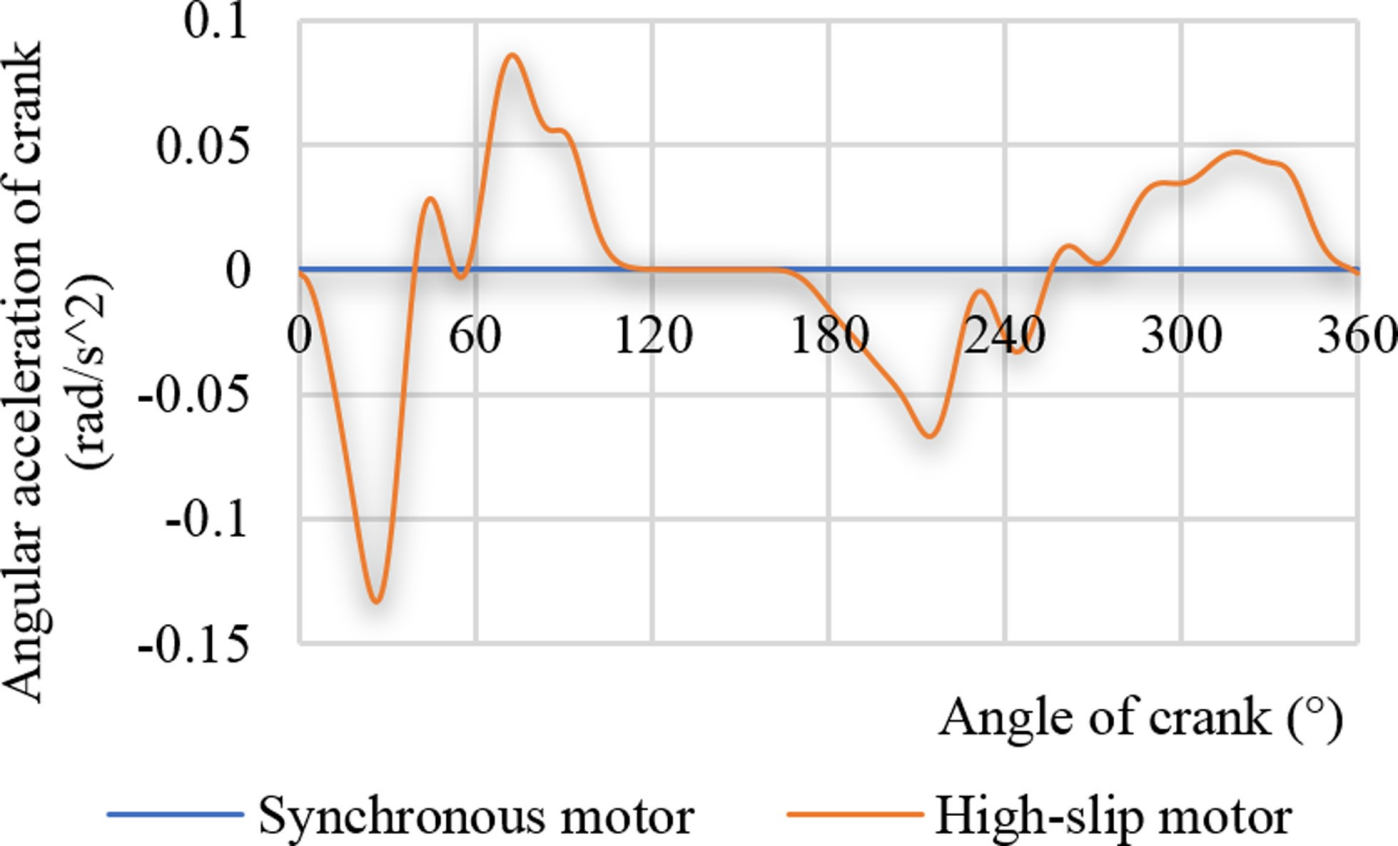
Angular acceleration curves of crank.

The two curves in Fig 5 are the angular acceleration curves obtained by solving the angular velocity of the crank in Fig 3 by the cubic spline quotient method under the driving conditions of the synchronous motor and the high-slip motor, respectively. The orange curve in Fig 5 represents the crank angular acceleration driven by the high-slip motor. The angular acceleration of the crank changes in a cycle and is determined by changes in the angular velocity of the crank. The crank angle acceleration is 0, while the synchronous motor is driven.

**Fig 6:**
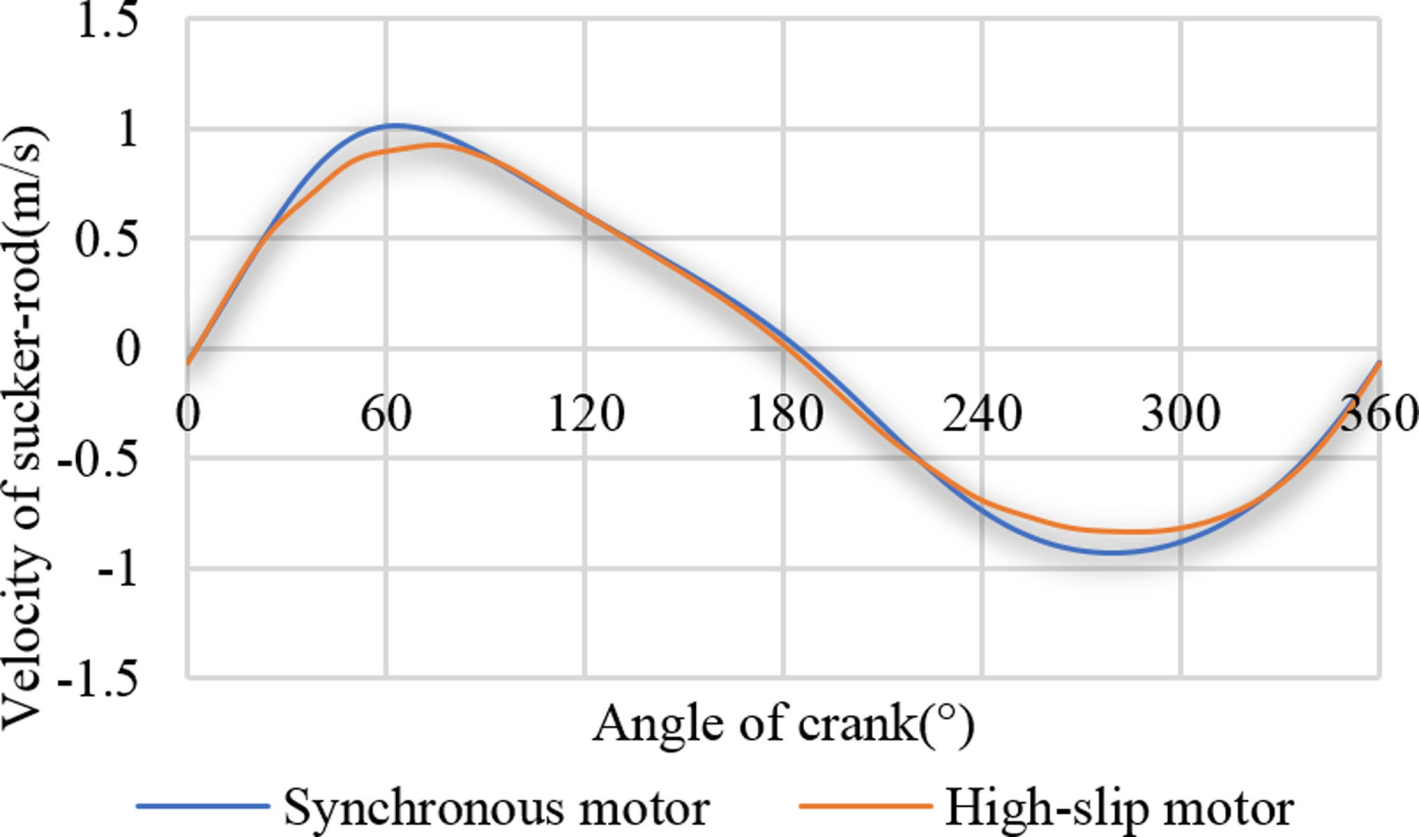
Velocity curves of the sucker rod.

Fig 6 represents two suspension speed curves driven by a synchronous motor and a high-slip motor. As presented in Fig 6, the high-slip performance of the high-slip motor affects the suspension speed curve. Comparing the torque curves, the peak value and the valley value of the net torque value corresponded to the peak value and the bottom point of the suspension velocity, respectively. When the synchronous motor is driven, the maximum suspension velocity is 1.01 m/s. When the high-slip motor is driven, the maximum suspension velocity is 0.93 m/s, which decreases 7.92%. The reduction in the suspension velocity is beneficial to reducing the suspension load.

**Fig 7:**
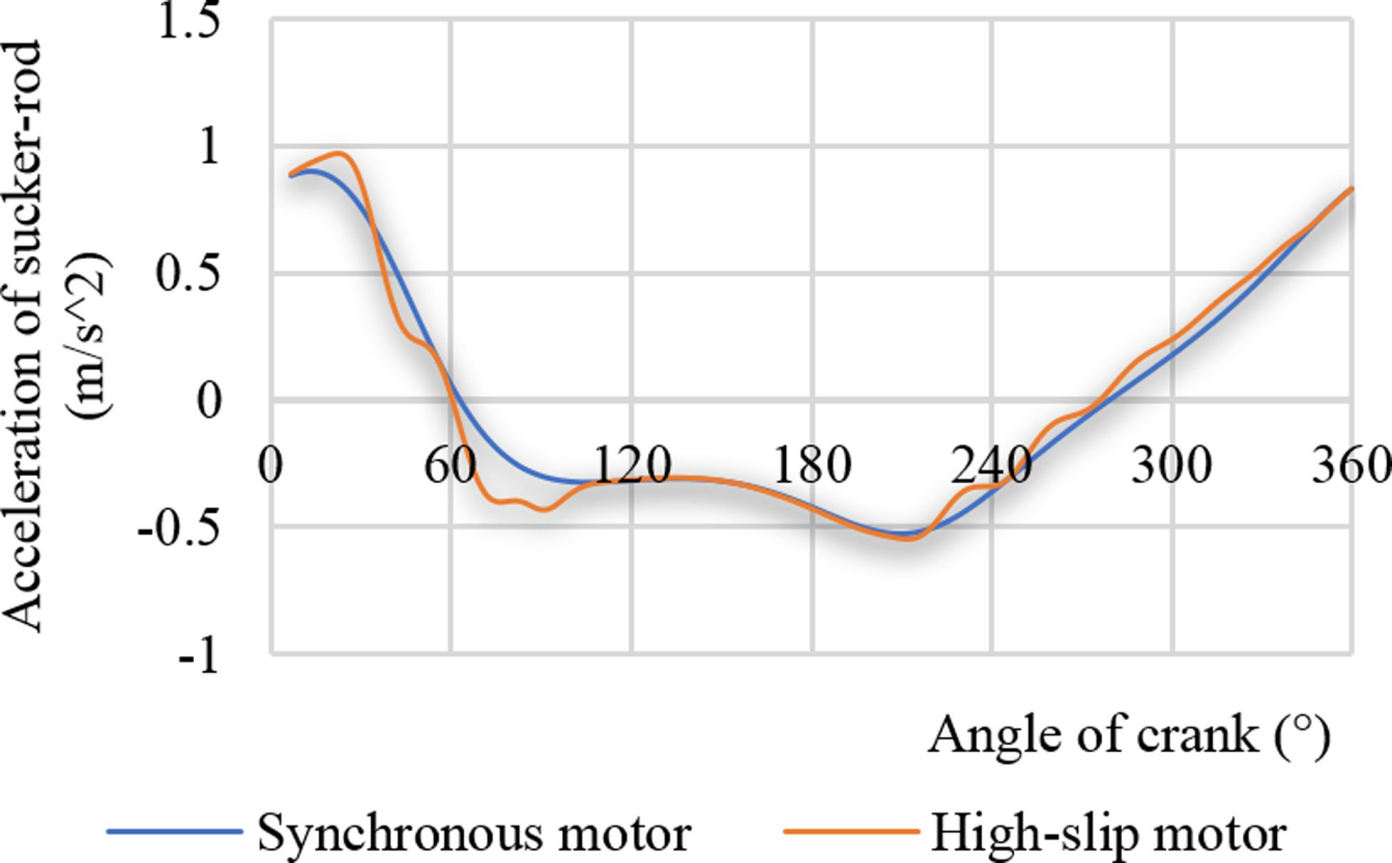
Acceleration curves of the sucker rod.

Fig 7 represents two suspension acceleration curves driven by a synchronous motor and a high-slip motor. The performance of the high-slip motor affects the suspension acceleration. The change rule of the suspension acceleration is more complicated. When the synchronous motor is driven, the maximum suspension acceleration is 0.90 m/s^2^. When the high-slip motor is driven, the maximum suspension acceleration is 0.97 m/s^2^, which is increased by 7.78%. Increasing the suspension acceleration increases the maximum suspension load, and Figs 6 and 7 show that the effect of the soft characteristic of the high-slip motor on the maximum suspension load is bidirectional. It is difficult to determine whether the high-slip ratio is favourable for reducing the maximum suspension load.

**Fig 8:**
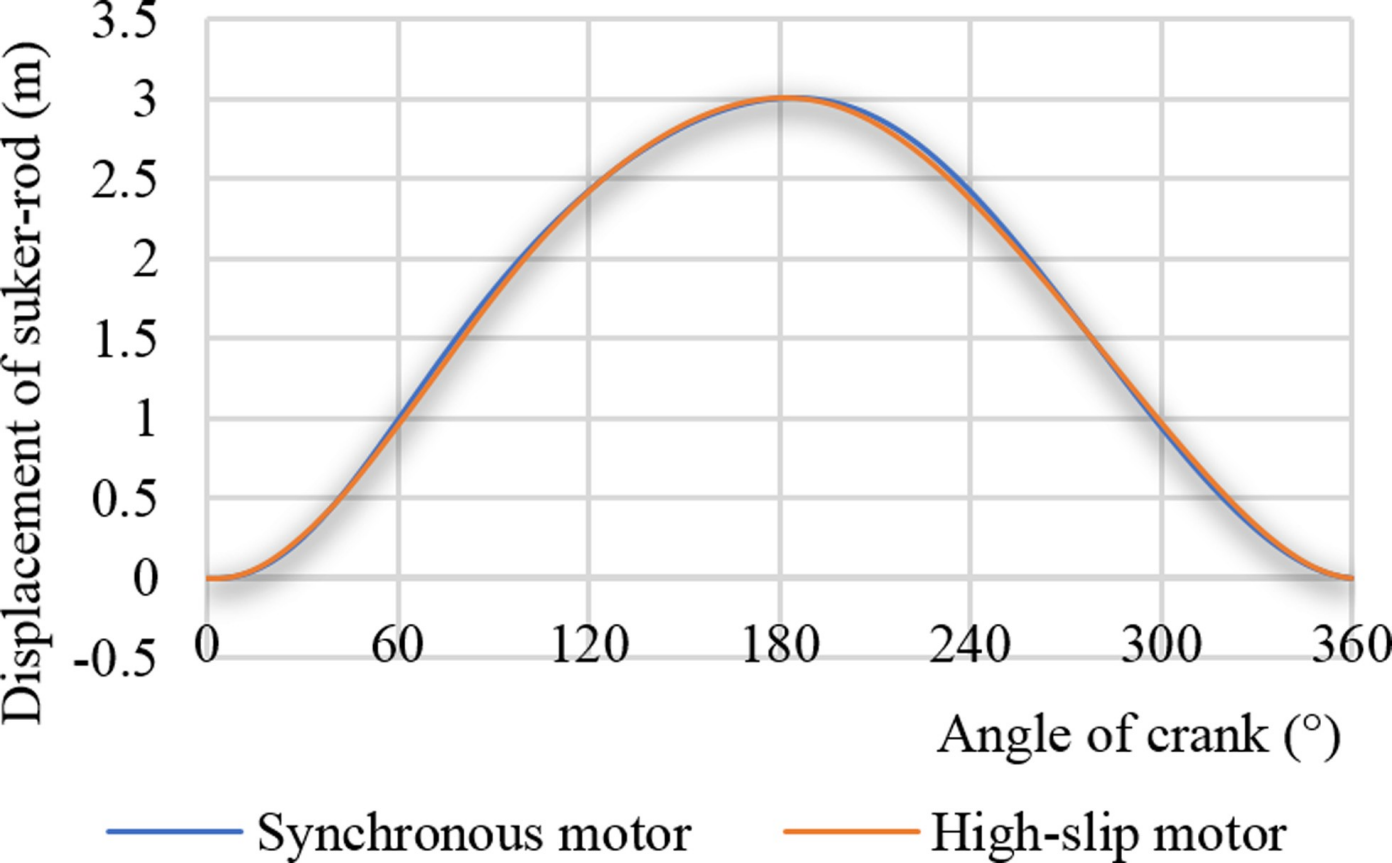
Displacement curves of the sucker rod.

Fig 8 represents the two suspension displacement curves driven by a synchronous motor and a high-slip motor. As shown in Fig 8, the performance of the high-slip motor has little effect on the suspension displacement curve.

**Fig 9:**
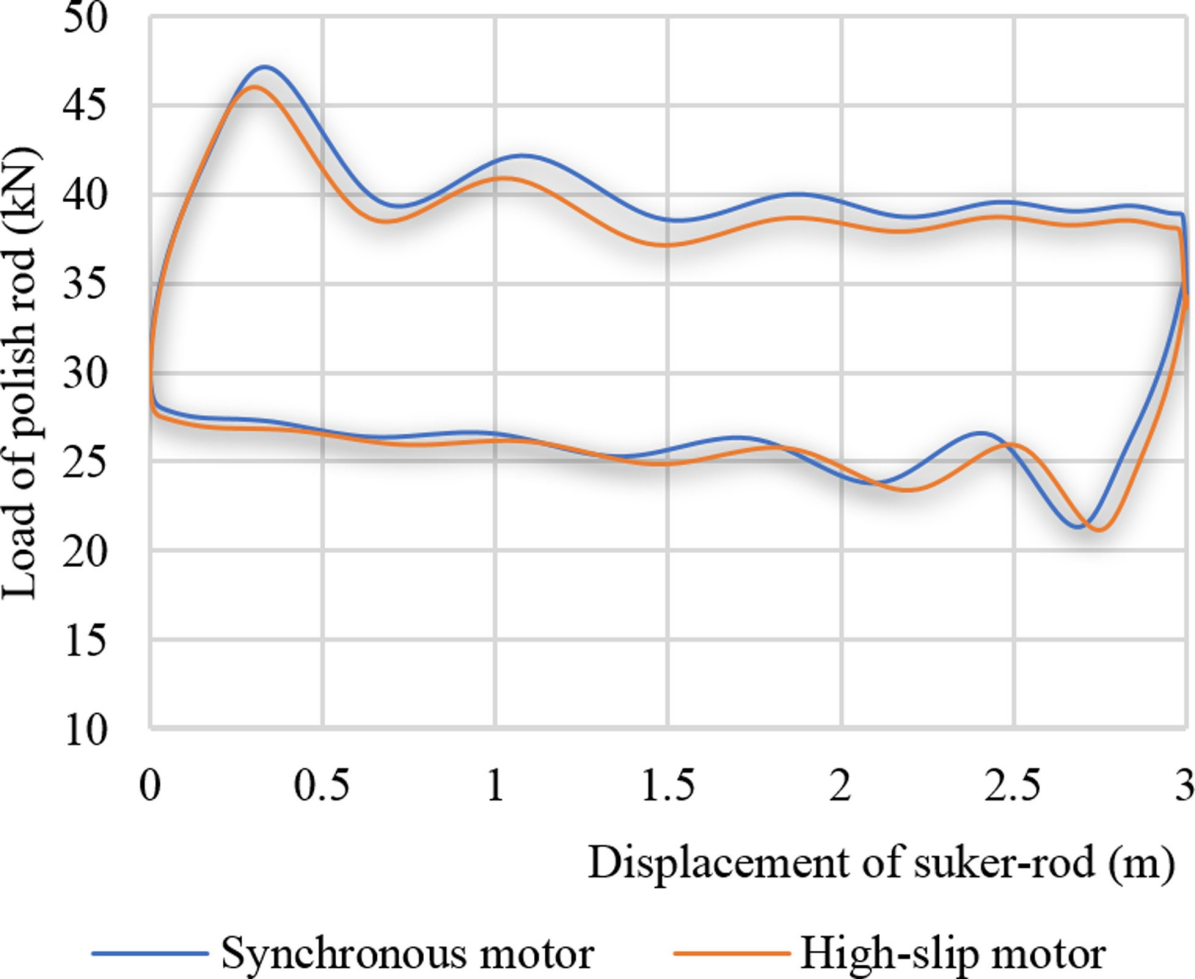
Load curves of the sucker rod.

The two curves in Fig 9 show the suspension point dynamometer diagram curves of the synchronous motor and the high-slip motor. When the synchronous motor is driven, the maximum suspension load was 47119 N. When the high-slip motor is driven, the maximum suspension load was 46010 N, and the reduction rate was 2.53%. The maximum suspension load reduction rate had no large magnitude of torque reduction because the effects of the suspension velocity and the suspension acceleration on the suspension load were opposing. The suspension load value is difficult to be judged based on the suspension velocity and the suspension acceleration. However, the soft characteristic of the high-slip motor has an effect on the suspension load, but the influence amplitude is relatively small.

**Fig 10:**
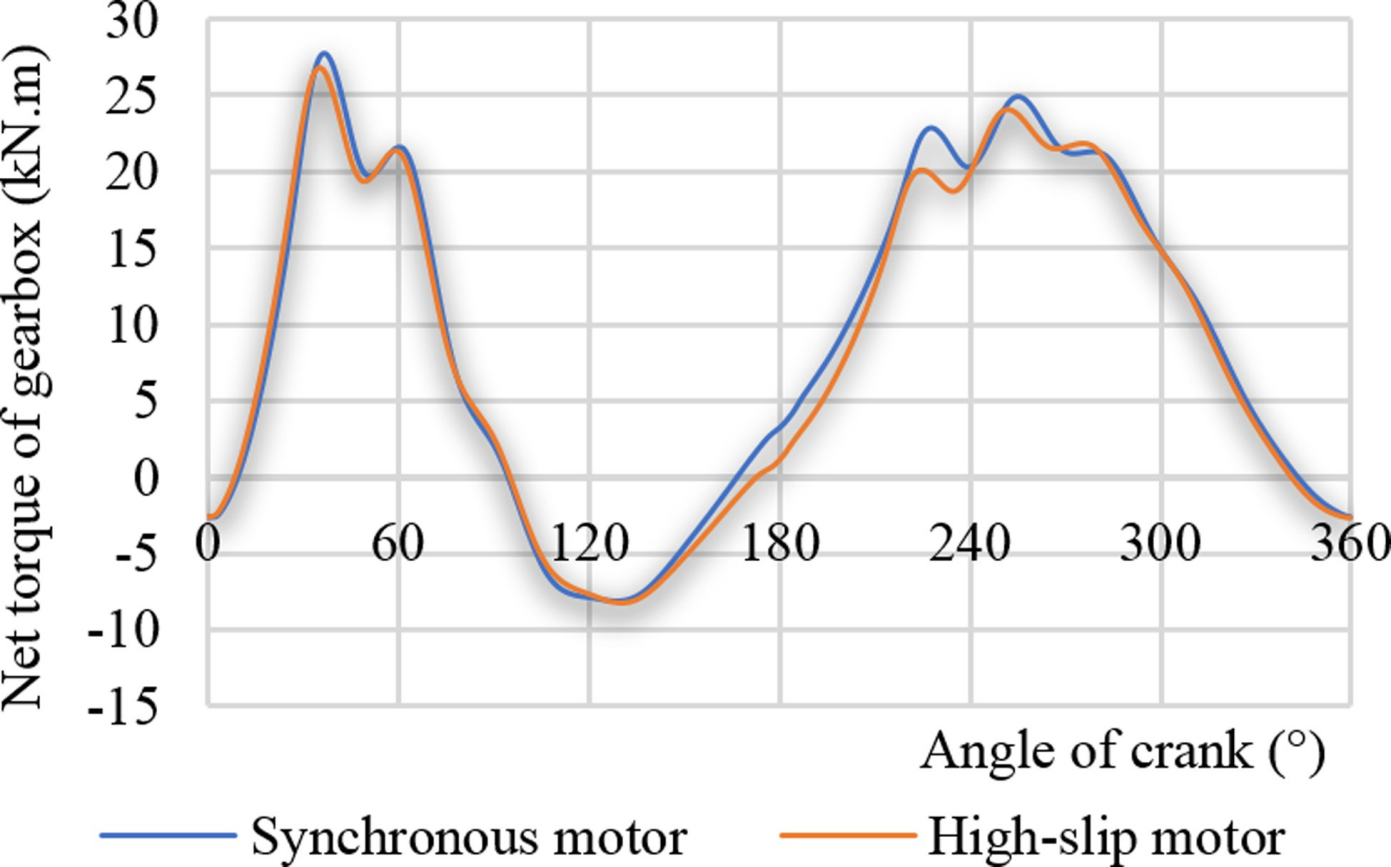
Net torque curves of the gearbox.

The two curves in Fig 10 are the net torque curves of the crank driven by the synchronous motor and the high-slip motor, respectively. When the synchronous motor is driven, the maximum net torque is 27734 N.m. When the high-slip motor is driven, the maximum net torque is 26840N.m, and the crank net torque is reduced by 3.2%, which this reduction is greater than the maximum suspension load reduction rate. This result also indicates that the equivalent rotation inertia *J*_*P*3_ on the crankshaft reduces the peak and volatility of the crank net torque curve. The soft characteristic of the high-slip motor has a positive effect on reducing the torque.

**Fig 11:**
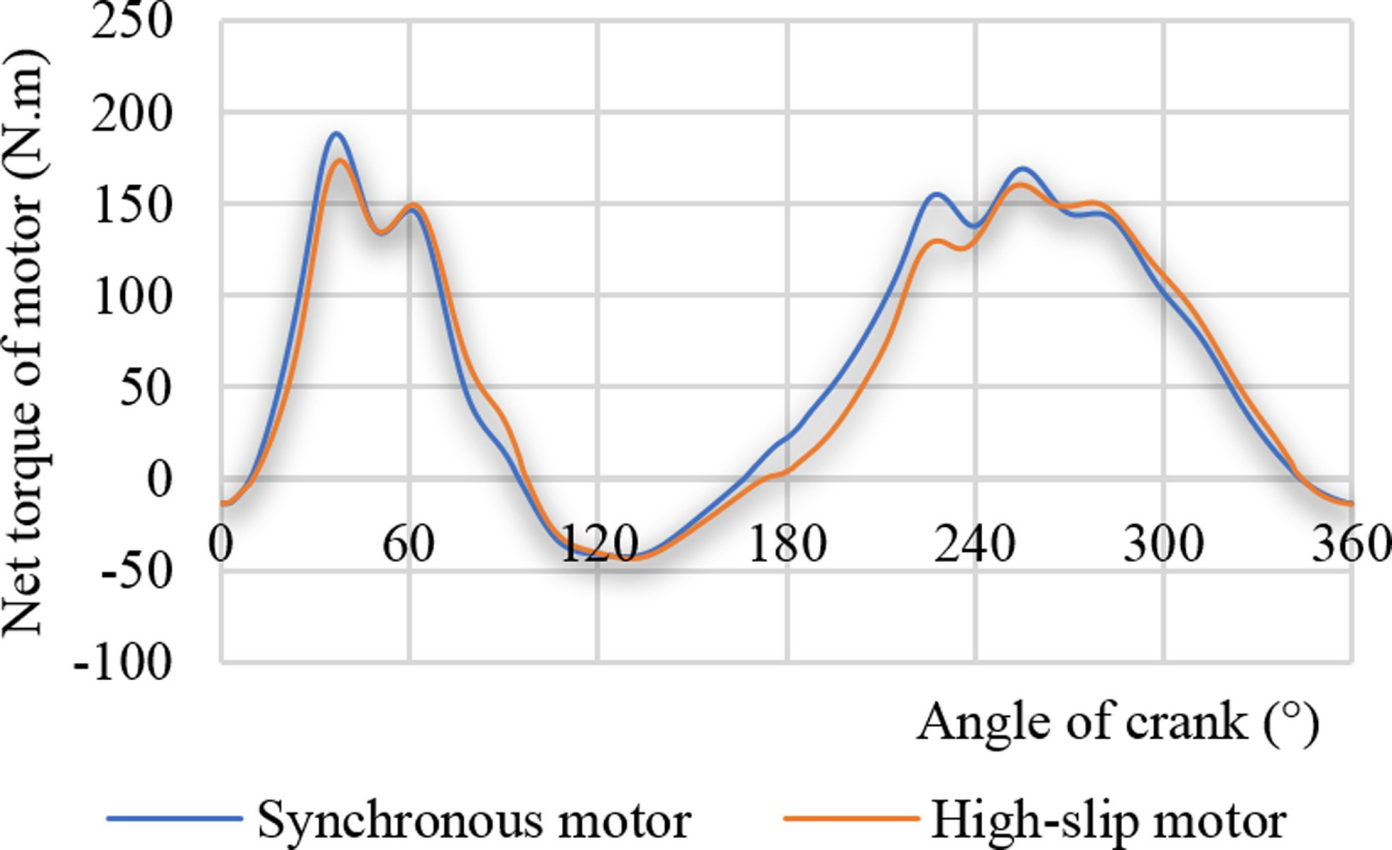
Net torque curves of motor.

The two curves in Fig 11 are the motor shaft torque curves driven by a synchronous motor and a high-slip motor, respectively. When the synchronous motor was driven, the maximum net torque of the motor was 188 N.m. When the high-slip motor is driven, the maximum shaft torque of the motor was 173 N.m, and the crank net torque was reduced by 7.98%, which this reduction is greater than the reduction rate of the net torque peak output of the gearbox. From the rotation inertia, the equivalent rotation inertia on the motor shaft is *J*_*p*_, and the equivalent rotation inertia on the crankshaft is *J*_*p*3_. The relationship between these two parameters is shown in Eq (26). In addition, Eq (26) shows that the motor shaft torque is more affected by the equivalent rotation inertia, also explaining the reason why the torque curve of the motor is less volatile. Reducing the volatility of the torque curve helps improve the efficiency of the motor.

When the synchronous motor is driven, the calculated cyclic load factors using the motor torque and the crank torque were 1.75 and 1.88, respectively. When the high-slip motor was driven, the calculated cyclic load factors using the motor torque and the crank torque were 1.92 and 2.17, respectively. The cyclic load factor represents the smoothness of the operation of the pumping unit system. Therefore, the soft characteristic of the high-slip motor can reduce the cyclic load factor, and this is conducive to the smooth running of the pumping well system. This result also shows that the cyclic load factors calculated by motor torque and crank torque are not the same as each other. The soft characteristic of high-slip motors has less influence on the motor, gearbox, and polished rod.

## B. Comparative analysis of experiments

In this experiment, the three-phase asynchronous motor (Y280S-8) with a rated power of 37 kW and the high-slip motor (YCHD280L) with a rated power of 37 kW were used to drive the beam pumping unit. Furthermore, a comparative analysis was performed. Under the same experimental conditions, the active power, reactive power, current and power factor of the two types of motors, output torque of the gearbox and suspension load were recorded accordingly.

The external characteristic equations of the tested motor (YCHD280L/37kw) are presented in Eqs 31–36.

Power factor:

$$\cos\phi {\left(p_{2}\right)}_{37\mathrm{kw}‐\mathrm{YCHD}}=-0.7878\times 0.959^{p_{2}}-0.0002p_{2}+0.855$$ (31)

Input power:

$$p_{1}{\left(p_{2}\right)}_{37\mathrm{kw}‐\mathrm{YCHD}}=0.0036p_{2}^{2}+0.9748p_{2}+3.305$$ (32)

Input electric current:

$$I_{1}{\left(p_{2}\right)}_{37\mathrm{kw}‐\mathrm{YCHD}}=0.0165p_{2}^{2}+0.5183p_{2}+55.286$$ (33)

Efficiency of the motor:

$$\eta {\left(p_{2}\right)}_{37\mathrm{kw}‐\mathrm{YCHD}}=88-88e^{\left(-p_{2}/6\right)}-0.0023p_{2}^{2}$$ (34)

Slip ratio:

$$s_{w}{\left(p_{2}\right)}_{37\mathrm{kw}‐\mathrm{YCHD}}=0.0008p_{2}^{2}+0.1066p_{2}$$ (35)

Mechanical characteristic:

$$n_{{}_{37\mathrm{kw}‐\mathrm{YCHD}}}=-6\times 10^{-6}T^{2}-0.0815T+750$$ (36)

**Fig 12:**
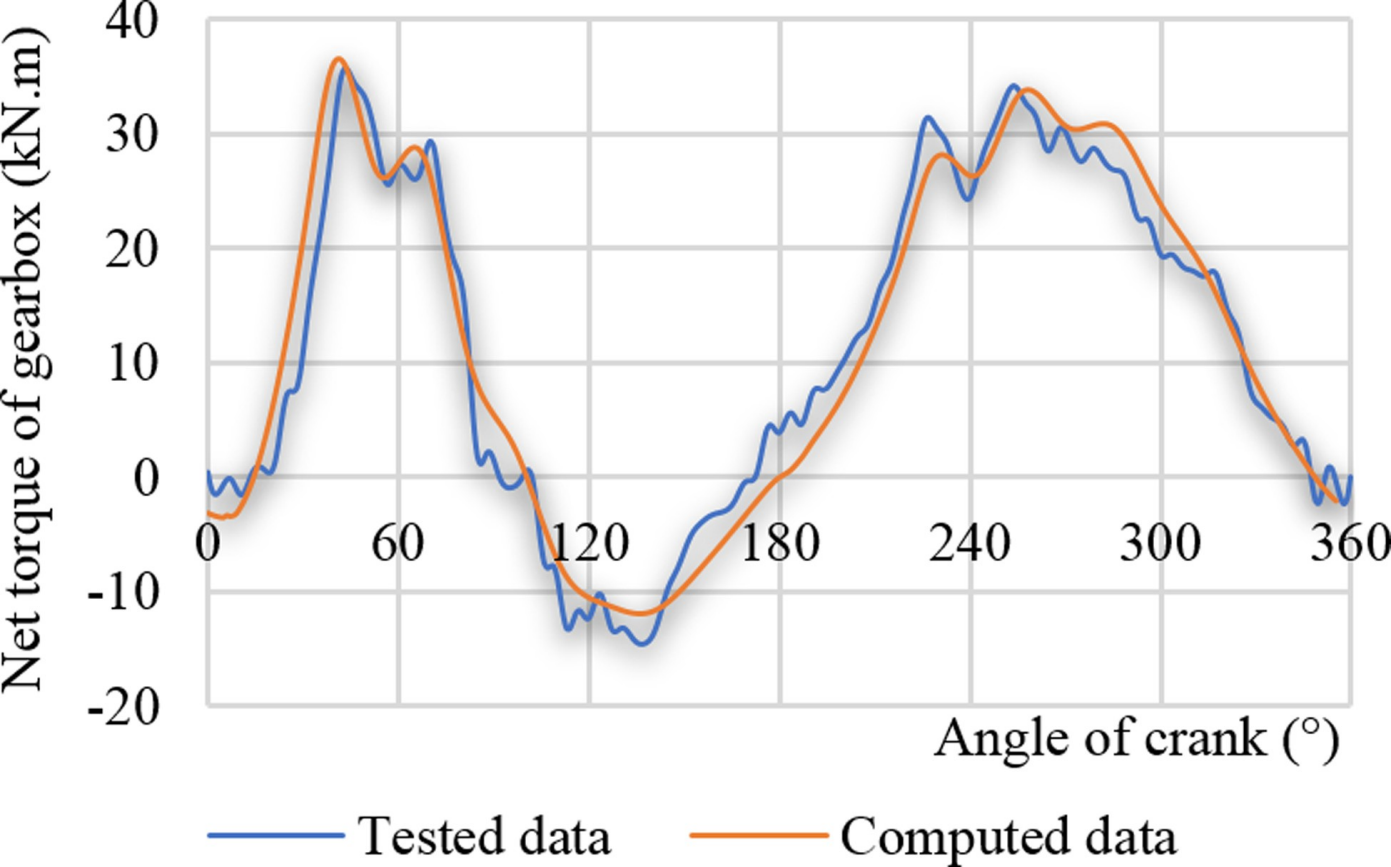
Net torque curves of the gearbox (YCHD280L-37 kW).

Table 3 shows the basic working data of the actual measurement when the two motors drove the pumping unit. The actual deviation is minor and can be used to compare and analyse the influence of the high-slip motor on the pumping well system. Fig 12 displays the test value and the theoretical calculation value of the net torque curve of the gearbox driven by the high-slip motor in the case of the actual measurement condition (see Table 4). As presented in Fig 12, the distribution trends of the two parameters are very similar. The tested and calculated torque maxima were 35917 N.m and 36457 N.m, respectively, with a deviation of 2.1%. The minimum tested torque and the minimum calculated torque were -14610 N.m and -12021 N.m, respectively, with a deviation of 17.7%. The average tested and calculated torque values were 11406 N.m and 11544 N.m, respectively, with a deviation of 1.2%. The maximum and average deviations of the tested and calculated torque were both less than 2.5%, and the deviation of the torque minimum was relatively large. The large deviation is because the calculation equation of the suspension load does not include the influence of the hole deviation, and the prediction error of the downstroke load is even larger. The presented analysis shows that the calculation method of this paper fully meets the needs of engineering calculation accuracy.

**Table 3:** Test conditions parameter test data.

Working motor	Measured stroke (m)	Measured impulse (1/min)	quality of balance	Measured liquid level (m)
**Y280S-8(37kW)**	3.02	5.9	0.93	600
**YCHD280L(37kW)**	2.99	6.04	0.96	605
**Deviation (%)**	1.0%	2.37%	3.22%	0.83%

**Table 4:** Comparison of test electrical parameters of two motors.

	Frequency of pump stroke (min^-1^)											
	9	6	4	9	6	4	9	6	4	9	6	4
**Type of motor**	**Averaged active power(kW)**			**Averaged reactive power(kW)**			**Averaged power factor** **(dimensionless)**			**Averaged current** **(A)**		
**Y280S**	14.13	8.14	5.94	22.46	20.16	20.25	0.52	0.38	0.28	50.07	36.87	34.01
**YCHD280L**	16.04	9.66	6.88	10.17	6.44	5.5	0.77	0.72	0.63	37.62	21.25	15.03
**Changing ratio (%)**	-13.52	-18.673	-15.82	54.72	68.06	72.84	48.08	89.47	125	24.87	42.37	55.81

**Fig 13:**
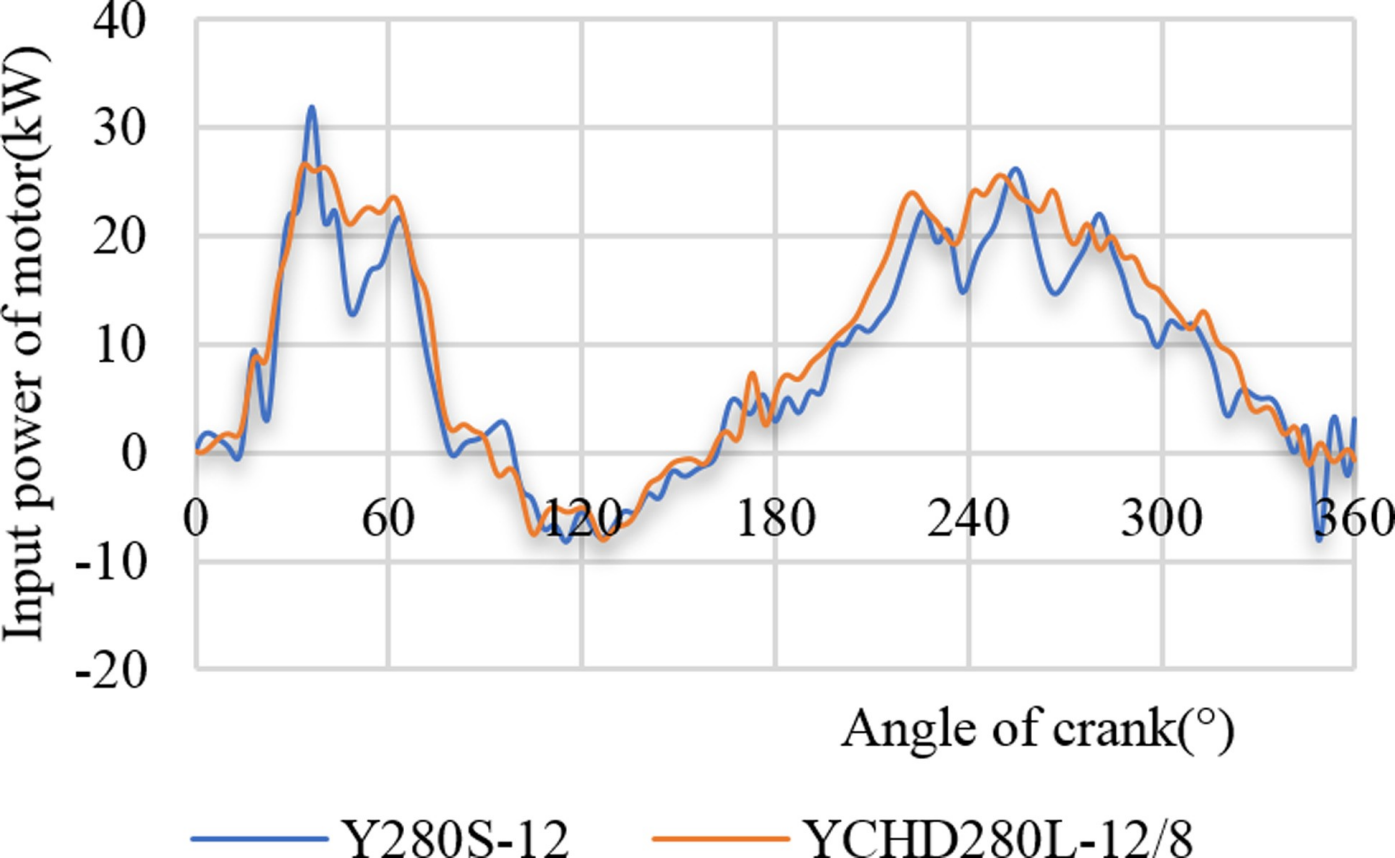
Comparison of two motor power curves.

Fig 13 shows two motor power curves at a stroke rate of 6 min^-1^, where the low-slip motor (Y280S-8) and the high-slip motor (YCHD280L) drove the pumping unit. Fig 13 shows that when the motor Y380S-8 drove the pumping unit, the high-frequency fluctuation part of the power curve is large and that the soft characteristic of the high-slip motor can weaken the high-frequency fluctuation part of the power curve. In addition, using this motor can also reduce the maximum power value. The power peaks of the low-slip motor and the high-slip motor are 31.85 kW and 26.60 kW, respectively, where the reduction rate is 16.48%.

Table 4 shows the comparison of the average values of the measured electrical parameters of the low-slip motor and the high-slip motor under the conditions of stroke rates of 9 min^-1^, 6 min^-1^, and 4 min^-1^. In Table 5, as the stroke frequency increases, the active power consumed by the high-slip motor increases. This increase is because the YCHD280L motor improves the slip rate by increasing the electrical resistivity. When the load rate of the motor increases, the active power increases naturally. Under the three kinds of stroke frequency conditions, the reactive power of the high-slip motor has been greatly reduced, all exceeding 50%, and the power factor of the high-slip motor has been greatly improved, all exceeding 60%. The average current of the high-slip motor has a large reduction, especially in the case of a stroke frequency of 4 min^-1^ where the reduction is 55%. By comparing and analysing the electrical parameters of the test, the high-slip motor has a positive effect on parameters such as reactive power, power factor and current, but the active power is increased by about 15%.

**Table 5:** Comparison of the output torque of the gearbox.

	Frequency of pump stroke (1/min)					
	9	6	4	9	6	4
**Type of motor**	Maximum value of net torque			Minimum value of net torque		
**Y280S-8**	48.24	36.99	31.54	-21.86	-15.62	-11.59
**YCHD280L-12/8**	44.15	35.72	33.15	-22.71	-14.61	-5.6
**Decreasing ratio of torque (%)**	8.48	3.44	-5.1	-3.9	6.47	51.68

**Fig 14:**
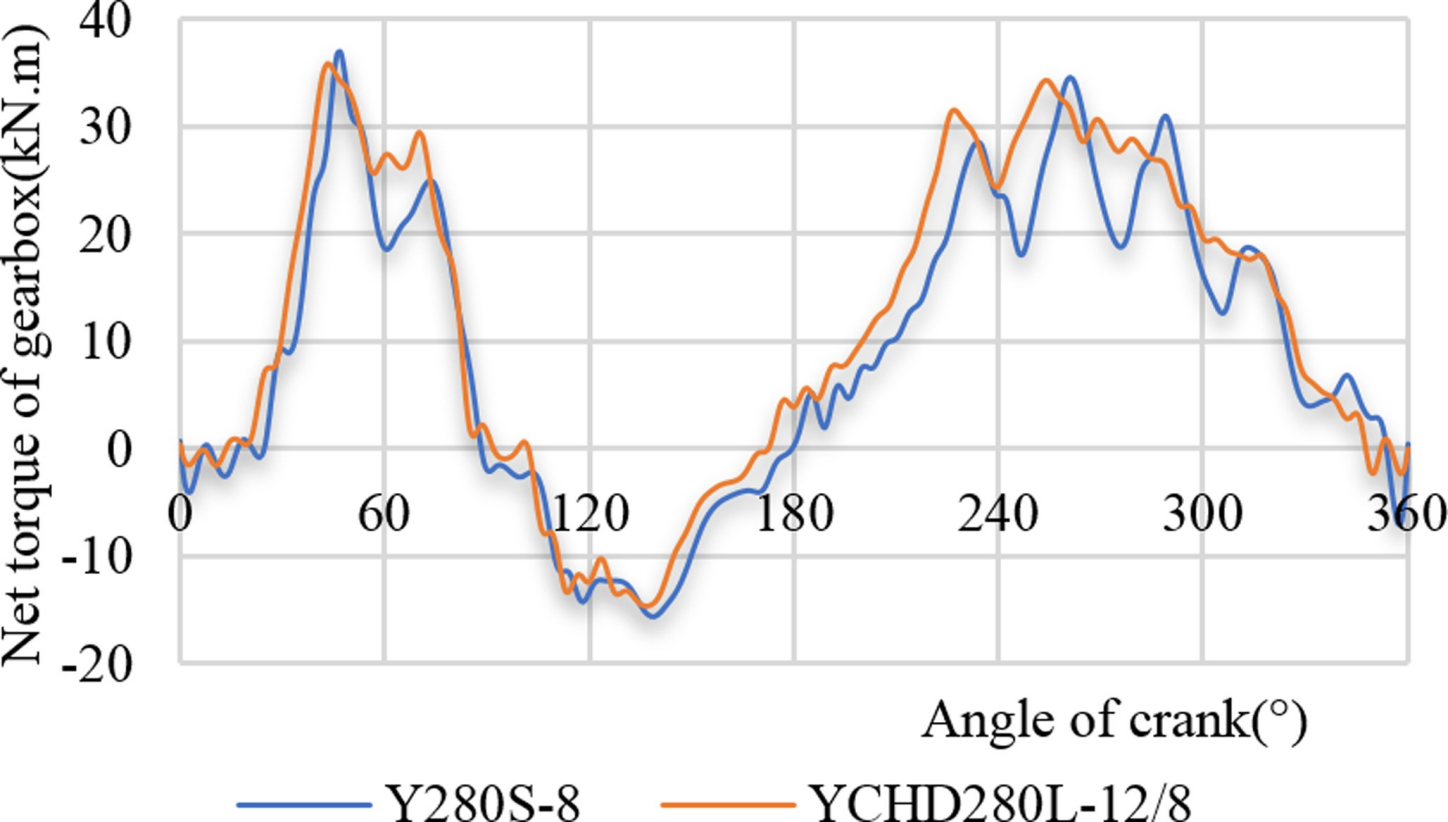
Comparison of output torque curve of the gearbox.

Fig 14 shows the net torque curves of the two gearboxes at a stroke frequency of 6 min^-1^, where the low-slip motor (Y280S-8) and the high-slip motor (YCHD280L) drove the pumping unit. Similar to the results presented in Fig 13, when the motor Y280s-8 drove the pumping unit, the high-frequency part of the torque curve fluctuated greatly. The soft characteristic of the high-slip motor can reduce the high-frequency part of the torque curve fluctuation and the maximum torque at the same time. When the low-slip motor and the high-slip motor drove pumping unit, the peak torques of the gearbox were 36.99 kW and 35.72 kW, respectively, where the reduction rate was 3.44%.

Table 5 shows the comparison of the gearbox casing torque peak and the torque valley while the low-slip motor (Y280S-8) and the high-slip motor (YCHD280L) were driven under the conditions of the stroke frequencies of 9 min^-1^, 6 min^-1^, and 4 min^-1^. As the stroke increased, the soft characteristic of the high-slip motor reduced the torque peak more greatly, and the torque peak decreased by 8.48% at a stroke frequency of 9 min^-1^. When the stroke frequency was less than 6 min^-1^, the soft characteristic of the high-slip motor helps reduce the negative torque. In the case of a stroke frequency of 9 min^-1^, the negative torque increased, which does not help the energy-saving and the smooth running of the pumping unit system.

## C. Comparison of dynamometer diagram

**Fig 15:**
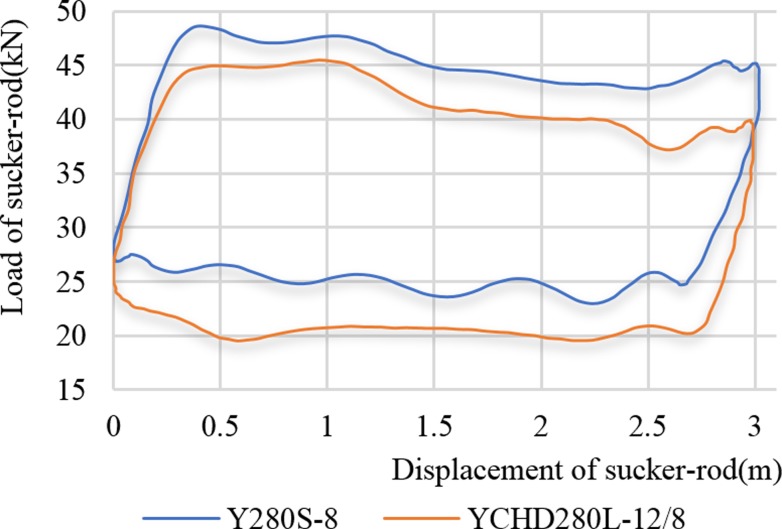
Comparison of the suspension dynamometer diagram curve at 6 times.

Fig 15 is a comparison of the dynamometer diagrams at a stroke rate of 6 min^-1^ while the low-slip motor (Y280S-8) and the high-slip motor (YCHD280L) drove the pumping unit. As Fig 15 shown, the high-slip motor reduced the suspension load more than the low-slip motor, with a reduction of 6.34%. From the overall view of the dynamometer diagram curve, the high-slip motor moves the dynamometer diagram downward. Comparing the maximum and minimum data of the suspension load in Table 6, the soft characteristic of the high-slip motor can reduce the suspension load and improve the safety of the polished rod.

**Table 6:** Comparison of suspended point loads.

	Frequency of pump stroke (1/min)					
	9	6	4	9	6	4
Type of motor	Maximum value of sucker-rod load			Minimum value of sucker-rod load		
**Y280S-8**	56.95	48.58	44.99	17	22.97	23.58
**YCHD280L-12/8**	52.92	45.5	43.76	15.58	19.55	18.65
**Decreasing ratio of sucker-rod load (%)**	7.02	6.34	2.73	8.35	14.89	19.27

Table 6 shows the comparison of the maximum and minimum suspended point loads while the low-slip motor (Y280s-8) and high-slip motor (YCHD280L) drove the pumping unit under the conditions of stroke rates of 9 min^-1^, 6 min^-1^ and 4 min^-1^. As the stroke increased, the maximum suspension load decreased. At a stroke rate of 9 min^-1^, the reduction rate was 7.02%. Under these three kinds of stroke conditions, the high-slip motor can also reduce the minimum suspension load.

## Conclusions

By motor testing, the external characteristics of the motor were regressed and had better precisions than presented in previous research. By combining the three-dimensional wave equation of the rod-tube-fluid, the kinematics equation of the four-bar mechanism and the differential equation of motor, the dynamic coupling model of the sucker-rod pump system was established. Then, we proposed a solution method of the dynamic coupling model between the pumping unit and high-slip motor with Visual Basic® V6.0 software.

Through the comparative analysis of the tested data and simulation results, the accuracy and practicability of the dynamic coupling model of pumping unit and high-slip motor were verified. The research results are as follows: The high-slip motor has soft characteristics that can decrease the fluctuation of the periodic load of the system, peak power of the motor, peak torque of the gearbox, and peak load of the polished rod. The high-slip motor also can reduce the high-frequency volatility of the motor power trace and the gearbox. Regarding the heavy oil wells, deep wells, polymer drive wells, and ternary composite drive wells, the high-slip motor is recommended for use. However, the high-slip motor is not suitable for the lower stroke wording condition and for lower-rated power pumping units.

The next research direction is recommended as follows:

1. Research the effect on the stroke by the high-slip motor, rod string, pump, submergence depth, the quality of balance.
2. Evaluate the effect of matching high-slip motors on other types of pumping units.
3. Explore the optimal design method between high-slip motors and pumping units.

## Supporting information

S1 Data (XLSX)
